# Laparoscopic common bile duct exploration through the cystic duct using flexible cholangioscopy combined with cholecystectomy for managing cholecysto-choledocholithiasis

**DOI:** 10.1055/a-2067-4587

**Published:** 2023-04-21

**Authors:** Wengang Zhang, Hui Ding, Zhenjuan Li, Enqiang Linghu

**Affiliations:** 1 Department of Gastroenterology, The First Medical Center of Chinese PLA General Hospital, Beijing, China; 2 Department of Gastroenterology, Henan Provincial People’s Hospital, Zhengzhou, Henan, China

Common bile duct (CBD) stones are present in approximately 3 % to 16 % patients with symptomatic gallstones 1 2 3 . Laparoscopic common bile duct exploration (LCBDE) and laparoscopic cholecystectomy (LC) could treat cholecysto-choledocholithiasis in one session. However, some drawbacks including the unsatisfactory controllability of the surgical choledochoscope, postoperative bile leak, and the use of the T-tube have hindered the further development of LCBDE + LC. To solve these problems, we introduced LCBDE through the cystic duct using a more flexible cholangioscope combined with cholecystectomy for cholecysto-choledocholithiasis.

A 59-year-old man with abdominal pain underwent computed tomography (CT) examination and the result showed cholecysto-choledocholithiasis. He chose LCBDE + LC to treat the gallstones and CBD stones in one session. During this procedure, we used a novel cholangioscope with flexible controllability, which was initially designed as a single-operator peroral cholangioscopy system 4 .

First the gallbladder, cystic duct, and cystic artery were dissociated under laparoscopy, and the cystic artery was cut off using the electrocoagulation function. Then a 5-mm incision was created on the cystic duct ( Fig. 1 a ). The cholangioscope was inserted into the cystic duct through the trocar with the help of the laparoscope ( Fig. 1 b ). Multiple CBD stones were found and extracted using a basket under cholangioscopy in multiple sessions ( Fig. 2 , Fig. 3 ). No residual stones were found under cholangioscopy and cholangiography ( Fig. 4 ). Finally, the cystic duct was clamped, and the gallbladder was removed ( Fig. 5 , Video 1 ). The patient’s recovery was smooth without any adverse events.

**Fig. 1:**
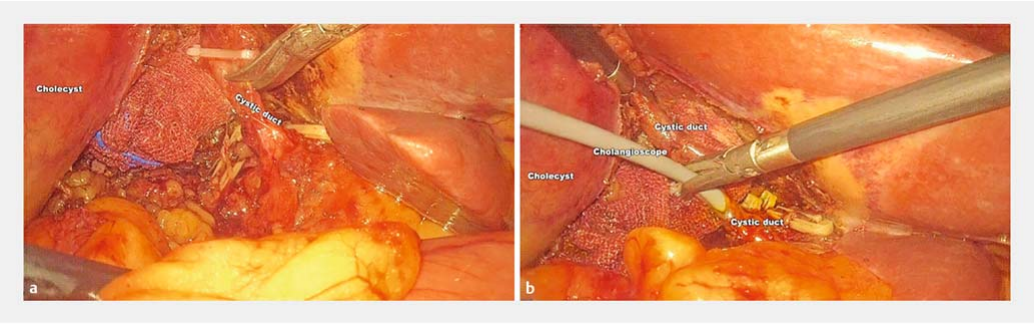
Laparoscopic image of common bile duct (CBD) exploration through the cystic duct. **a** A 5-mm incision was created on the cystic duct. **b** The cholangioscope was inserted into the cystic duct through the trocar with the help of the laparoscope.

**Fig. 2:**
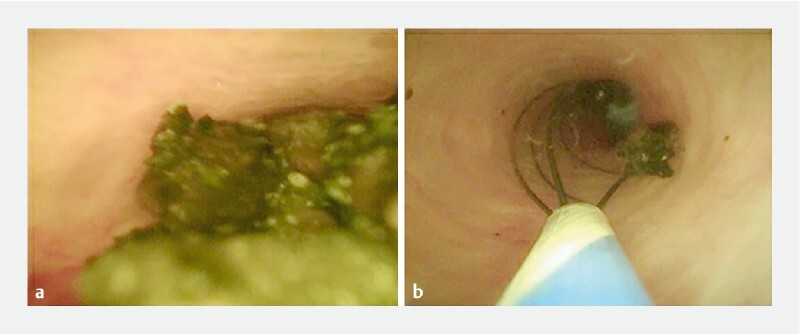
Cholangioscopy image of common bile duct exploration through the cystic duct. **a** Multiple CBD stones were found. **b** CBD stones were extracted using a basket.

**Fig. 3:**
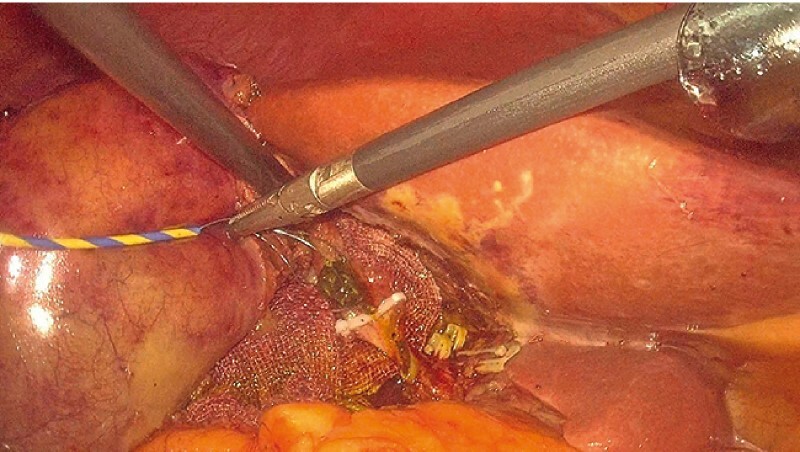
CBD stone was removed from the cystic duct under laparoscopy.

**Fig. 4:**
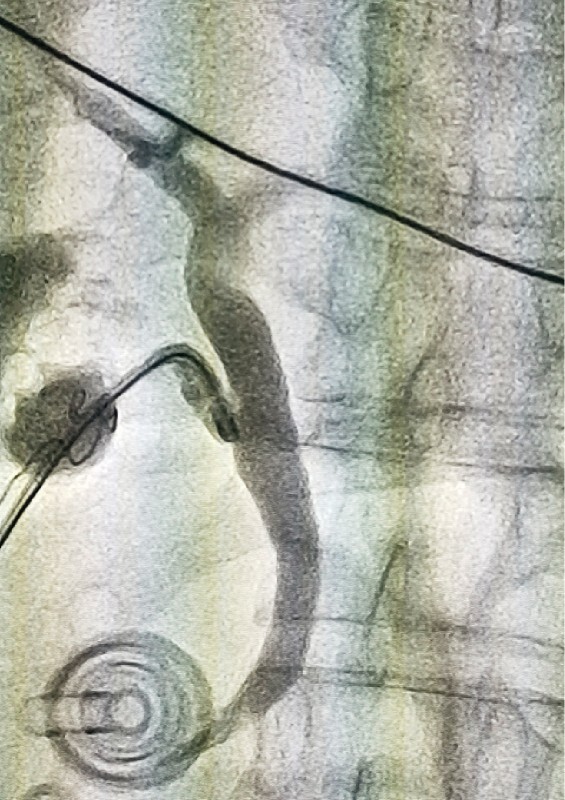
No residual stones were found under cholangiography.

**Fig. 5:**
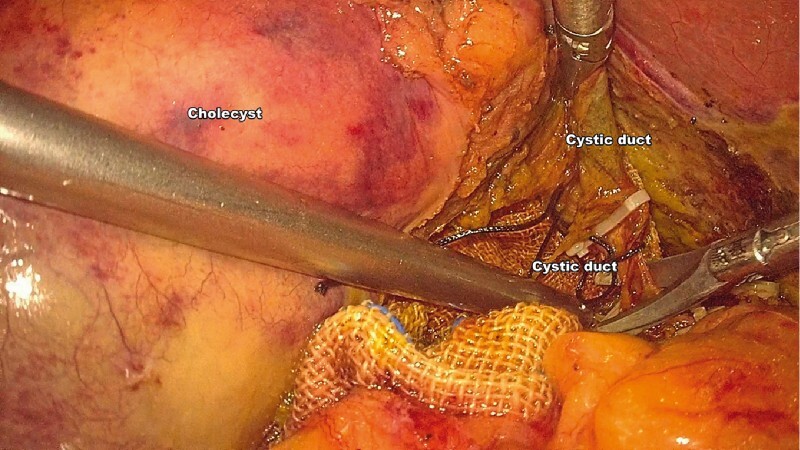
The cystic duct was clamped, and the gallbladder was removed.

**Video 1** Laparoscopic common bile duct exploration through the cystic duct using flexible cholangioscopy combined with cholecystectomy.


Of note, this is the first experience of this technique in our team, and we hope that patients with cholecysto-choledocholithiasis can benefit from this procedure if conditions allow.

The advantages of this technique over traditional LCBDE + LC include better controllability, enabling complete clearance of CBD stones, and a smaller incision in the cystic duct instead of the CBD, avoiding the placement of a T-tube and postoperative bile leak.

Endoscopy_UCTN_Code_TTT_1AR_2AH

## Notes

Endoscopy E-Videos

https://eref.thieme.de/e-videos
*E-Videos* is an open access online section of the journal *Endoscopy* , reporting on interesting cases and new techniques in gastroenterological endoscopy. All papers include a high-quality video and are published with a Creative Commons CC-BY license. Endoscopy E-Videos qualify for HINARI discounts and waivers and eligibility is automatically checked during the submission process. We grant 100% waivers to articles whose corresponding authors are based in Group A countries and 50% waivers to those who are based in Group B countries as classified by Research4Life (see: https://www.research4life.org/access/eligibility/ ). This section has its own submission website at https://mc.manuscriptcentral.com/e-videos
